# BiOBr nanoparticle-modified Ti_3_C_2_T_*x*_ MXenes for photocatalytic degradation of organic arsenic in wastewater

**DOI:** 10.1039/d5ra02929e

**Published:** 2025-06-23

**Authors:** Yaxin Guo, Ya-Nan Wang, Jinsong Peng, Haiyan Song, Chunxia Chen

**Affiliations:** a Department of Chemistry and Chemical Engineering, College of Chemistry, Chemical Engineering and Resource Utilization, Northeast Forestry University Harbin 150040 China chem_shy@163.com ccx1759@163.com; b Center for Innovative Research in Synthetic Chemistry and Resource Utilization, Northeast Forestry University Harbin 150040 China

## Abstract

Arsenic (As) contamination in water remains a serious concern due to its high toxicity and harmful effects on human health and the environment. Herein, we successfully synthesized a novel photocatalyst (BiOBr/Ti_3_C_2_) by *in situ* inserting BiOBr nanoparticles into Ti_3_C_2_T_*x*_ MXenes for the photocatalytic degradation of organic arsenic roxarsone (3-nitro-4-hydroxyphenylarsonic acid) in wastewater. BiOBr/Ti_3_C_2_ exhibited a unique morphology characterized by uniform BiOBr nanoparticles within more dispersed Ti_3_C_2_T_*x*_ layers. Heterojunctions were formed between Ti_3_C_2_T_*x*_ and BiOBr, which were conducive to photogenerated charge separation and electron transfer in Ti_3_C_2_T_*x*_ layers. An optimal BiOBr/Ti_3_C_2_ photocatalyst achieved a removal efficiency of 1.27 mg g_cat._^−1^ h^−1^ for 2 mg L^−1^ of roxarsone wastewater within 0.5 h and a higher removal rate (about 1.5 times at 3 h) than pure Ti_3_C_2_T_*x*_. In addition, BiOBr/Ti_3_C_2_ exhibited an apparent quantum yield (AQY) of 29.5% and good reusability for 4 cycles. The enhanced photocatalytic performance was mainly attributed to the intercalation of BiOBr nanoparticles within Ti_3_C_2_T_*x*_ layers, which increased reaction space and improved the separation and transport of photocarriers. Holes (h^+^) and ˙OH in the valence band (VB) of BiOBr/Ti_3_C_2_ were involved in the main route of roxarsone oxidative mineralization. The present BiOBr/Ti_3_C_2_ system provides fundamentals for the sustainable photocatalytic treatment of wastewater containing organic arsenic.

## Abstract 2

BiOBr NPs@Ti_3_C_2_T_*x*_ for photocatalytic degradation of roxarsone.

## Graphical abstract

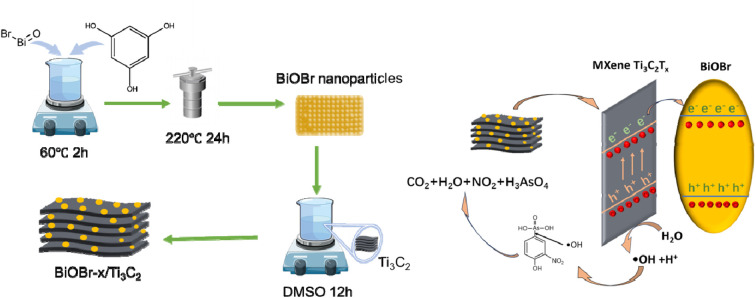

## Introduction

Roxarsone is an organic arsenic feed additive that has been widely used in the poultry and pig industries for decades to promote animal growth and treat coccidiosis.^1^ A portion of the roxarsone added to the feed cannot be absorbed by poultry and pigs, and eventually remains in the faeces. Since roxarsone is highly water-soluble, it is easily transferred from animal feces to the surrounding aqueous environment, such as surface water or groundwater.^2–6^ Organic arsenic has low toxicity, but in the natural environment, it can be decomposed into highly toxic inorganic arsenic through biological or photooxidation reactions.^7–9^ Furthermore, inorganic arsenic can be divided into As(iii) and As(v).^10^ As(iii) and As(v) can be rapidly absorbed from the gastrointestinal tract, causing inevitable damage to the human body, further increasing their potential risks to human health and ecology.^3,11–16^ Aromatic amine feed additives, including organic arsenic, have been banned worldwide because of the concerns about arsenic contamination in the environment.^2,17^ However, after decades of use, organic arsenic has been accumulated in large quantities in the environment. The degradation, transformation, migration and impact of organic arsenic on organisms have attracted the attention of many countries around the world. At present, the main reported organic arsenic removal technologies are categorized as biological methods,^18–21^ adsorption,^22–26^ and advanced oxidation processes such as UV-based processes,^27–30^ sulfite (S(iv)) oxidation processes,^31^ and Fenton/Fenton-like processes.^32–34^ However, these techniques have some limitations. The biological degradation of organic arsenic is slower and may produce more toxic alkyl arsenic compounds.^18^ Adsorption often requires long contact times and high costs.^22,23^ Advanced oxidation processes are introduced into the treatment of arsenic-containing water. As an advanced oxidation process, photocatalytic oxidation is widely used in the oxidative removal of organic or inorganic pollutants because it can use clean and pollution-free light energy,^35^ produce a large number of strong oxidizing active substances and have no secondary pollution. However, highly toxic inorganic arsenic compounds will cause serious pollution to the environment, in case the inorganic arsenic compounds produced after oxidation are not treated reasonably in time. Therefore, the current photocatalysis technology has been widely considered and put into practice. The problems of current photocatalytic systems for the degradation of organic arsenic include the low utilization of visible light and the fast charge recombination speed, which limit their applications. Therefore, various photocatalysts, such as TiO_2_,^36^ ZnO,^37^ CuO^38^ and goethite^39^ were developed to enhance the photodegradation of organic arsenic. Up to now, the MXene family has not been reported in the photocatalytic degradation of organic arsenic.

MXenes are a very famous family of two-dimensional (2D) materials,^40^ and have been widely used in photocatalysis, batteries and supercapacitors.^41–43^ Ti_3_C_2_T_*x*_, as a member of MXenes, has excellent electron transportability, a large specific surface area and adjustable active sites, which may promote the separation and transport of photoexcited electron–hole pairs.^44^ The construction of composites based on Ti_3_C_2_T_*x*_ is very simple because of the flexibility and unique layered structure of Ti_3_C_2_T_*x*_. In Ti_3_C_2_T_*x*_ composites, Ti_3_C_2_T_*x*_ not only increases the absorption of light but also causes rapid carrier migration and inhibits its recombination.^45^ Therefore, Ti_3_C_2_T_*x*_ is often used as a noble metal^41^ co-catalyst to improve the photocatalytic activity of complexes.^40,46–48^ It has also been noted that Ti_3_C_2_T_*x*_ with –F and –OH end groups, originating from exfoliation with HF, possess a narrow bandgap and also have potential photocatalytic properties.^49^ However, the photocatalytic performance of MXene Ti_3_C_2_T_*x*_ was not satisfactory. Its application is limited by rapid charge recombination. Various methods have been developed to overcome this problem. MXenes have been coupled to various semiconductors (such as TiO_2_ and g-C_3_N_4_) to form heterojunctions, thereby facilitating photogenerated electron–hole separation and improving light absorption in photocatalyzed reactions. Considering that the properties of 2D accordion-like MXene Ti_3_C_2_T_*x*_ can be improved by changing the elemental composition and adjusting the surface functional groups, it is an effective approach to introduce heteroatoms to modify the surface and improve the photocatalytic property.

Currently, many efficient photocatalyst materials have emerged.^50,51^ Among them, BiOBr is a typical bismuth oxyhalide material (BiOX, X = Cl, Br, I) that has been widely used in photocatalysis because of its suitable energy band structure and chemical stability.^52^ However, the photocatalytic performance of BiOBr is limited by the low separation efficiency of the charge carriers. Therefore, various strategies have been adopted to improve its photocatalytic performance,^53^ such as bismuth-rich strategy, element doping,^50,51,54,55^ defect engineering and heterostructure construction.^56^ Coupling with other semiconductors is one of the most common ways to accelerate the separation efficiency of photogenerated electron–hole pairs. It was found that there are almost no reports on BiOBr nanoparticle-modified MXene Ti_3_C_2_T_*x*_ materials, even their use in the photocatalytic degradation of organic arsenic.

In this work, we successfully synthesized a novel composite of BiOBr nanoparticle-modified MXene Ti_3_C_2_T_*x*_, which was used in the photocatalytic removal of roxarsone from wastewater. A heterojunction was attempted to establish between MXene Ti_3_C_2_T_*x*_ and BiOBr nanoparticles to improve the degradation efficiency of roxarsone. The ultra-fine and highly dispersed BiOBr nanoparticles effectively expanded the specific surface area, enhanced light absorption, and promoted carrier separation on the Ti_3_C_2_T_*x*_ matrix. Compared with pure Ti_3_C_2_T_*x*_, the degradation efficiency of roxarsone in the BiOBr/Ti_3_C_2_ composite was significantly enhanced. A mechanistic study revealed that the holes (h^+^) and ˙OH in the VB of BiOBr/Ti_3_C_2_ primarily contributed to the oxidative mineralization of roxarsone.

## Experimental

### Chemicals

MXene Ti_3_C_2_T_*x*_ was purchased from Jilin Yiyi Technology Co., Ltd. KBr and C_2_H_5_OH were purchased from Tianjin Tianli Chemical Reagent Co., Ltd. Bi(NO_3_)_3_·5H_2_O and 4-hydroxy-2,2,6-tetramethylpiperidinyloxy (TEMPOL) were purchased from Macklin Chemical Co., Ltd. Dimethyl sulfoxide (DMSO) was purchased from Tianjin Fuyu Fine Chemical Co., Ltd. Phloroglucinol (C_6_H_6_O_3_) was purchased from Tianjin Fuchen Co., Ltd. Ethylenediaminetetraacetic acid (EDTA) was purchased from Tianjin Beilian Fine Chemicals Development Co., Ltd. Isopropyl alcohol (IPA) was purchased from Aladdin Co., Ltd.

### Preparation of BiOBr

0.669 g of KBr was dissolved in 75 mL of deionized water and heated to 90 °C. 2.429 g of Bi(NO_3_)_3_·5H_2_O was dissolved in 100 mL of C_2_H_5_OH by ultrasonication to form a suspension. KBr solution was added to the suspension. After 30 min of the reaction, a pale yellow precipitate was obtained. The yellow precipitate was collected by centrifugation, washing, and drying at 60 °C for 12 h to obtain BiOBr.

### Preparation of BiOBr nanoparticles

1.07 g of the as-prepared BiOBr and 0.27 g of C_6_H_6_O_3_ were dissolved in 40 mL of deionized water, stirred at 60 °C for 2 h, and then subjected to hydrothermal treatment at 200 °C for 12 h. The obtained product was washed 3 times with water and ethanol and dried at 60 °C for 6 h to obtain BiOBr nanoparticles.

### Preparation of BiOBr/Ti_3_C_2_

0.05 g of MXene Ti_3_C_2_T_*x*_ and the required masse of BiOBr nanoparticles were dispersed into 40 mL of DMSO, stirred at room temperature for 12 h, and then treated by ultrasonication for 1 h. After centrifugation, washing with water and ethanol for 3 times, and drying at 60 °C for 8 h, a series of products labeled as BiOBr-*x*/Ti_3_C_2_ (*x* wt% = 5 wt%, 10 wt%, 20 wt% loading of BiOBr nanoparticles) was finally obtained.

### Characterizations

X-ray diffraction (XRD) patterns were recorded using a Rigaku D/Max 2400 diffractometer with Cu Kα radiation. Fourier transform infrared (FT-IR) spectroscopy was performed on a Nicolet Thermo 360 spectrometer using the KBr pellet method. Scanning electron microscopy (SEM) was performed using a JSM-7500 F field emission SEM (JEOL, Japan). Transmission electron microscopy (TEM) images were obtained using a FEI Tecnai G2 F20 field emission transmission electron microscope. X-ray electron spectroscopy (XPS, THERMO) was used to analyze the composition and electronic states of the elements, and the scanning spectra of Ti 2p, C 1s, Bi 4f, Br 3d and O 1s were obtained. Photocurrent response and electrochemical impedance spectroscopy (EIS) were performed on an electrochemical workstation (CHI650D, China) with a 300 W xenon lamp and a 420 nm cut-off filter as the visible light source. UV-vis diffuse reflectance spectra were obtained on a JASCO V-770 spectrometer with BaSO_4_ as the internal standard. Photoluminescence (PL) spectroscopy and time-resolved photoluminescence (TRPL) were performed using a fluorescence spectrometer (Edinburgh Instrument FLS980) with an excitation wavelength of 373 nm.

### Photocatalytic activity tests

50 mg of the photocatalyst sample was dispersed in 50 mL of roxarsone aqueous solution containing 2 mg L^−1^ of the initial concentration (unless otherwise noted). The system temperature was controlled at 0 °C using a water bath and was operated under continuous stirring. Before photoreaction, the reactant was controlled in the dark to reach the adsorption–desorption equilibrium. A 300 W xenon lamp with a cut-off filter of 420 nm was used as the visible light source. The suspension was then sampled and centrifuged at the required time interval. The absorbance of roxarsone was measured using the UV-vis spectrophotometer. Eqn (1) was used to calculate the removal efficiency of roxarsone. The liquid residual inorganic products of roxarsone after photocatalytic degradation were analyzed by high-performance liquid chromatography-inductively coupled plasma mass spectrometry LC-ICP-MS (LC, Prin-Cen, Elspe-2; ICP-MS, NeXion1000 G, PerkinElmer, USA). By changing the loading of BiOBr on Ti_3_C_2_T_*x*_, the reaction temperature, pH value and initial concentration of roxarsone were determined according to the same procedure. The recycle test was also performed following the same procedure in 1 h using the regenerated photocatalyst in every cycle. By adding 2 mmol of the scavengers, a trapping experiment was carried out to study the radical contribution, during which EDTA, DMSO, IPA and TEMPOL were used as scavengers of h^+^, e^−^, ˙OH and ˙O_2_^−^, respectively. The apparent quantum yields (AQY) of the photocatalytic degradation of roxarsone were measured using a monochromatic lamp with bandpass filters at 320, 450, 550, 600 and 750 nm. The irradiation intensity was measured as 10.95 W m^−2^ using a light power detector (SM206-SOLAR). The irradiation area was controlled at 13.5 cm^2^. The AQY of the photocatalytic degradation of roxarsone for 1 h was calculated using eqn (2).

1 *D* = (*C*_0_ − *C*_*t*_)/*C*_0_ = (*A*_0_ − *A*_*t*_)/*A*_0_

where *D* is the removal efficiency, *C*_0_ is the initial concentration of roxarsone, *C*_*t*_ is the concentration of roxarsone at *t* min, *A*_0_ is the absorbance of roxarsone at the initial concentration, *A*_*t*_ is the absorbance of roxarsone at *t* min.

2 AQY (%) = (*N*_e_/*N*_p_) × 100%

where *N*_e_ is the total number of electrons transferred during the reaction and *N*_p_ is the number of incident photons.

## Results and discussions

### Morphology and structure of materials

**Scheme 1:**
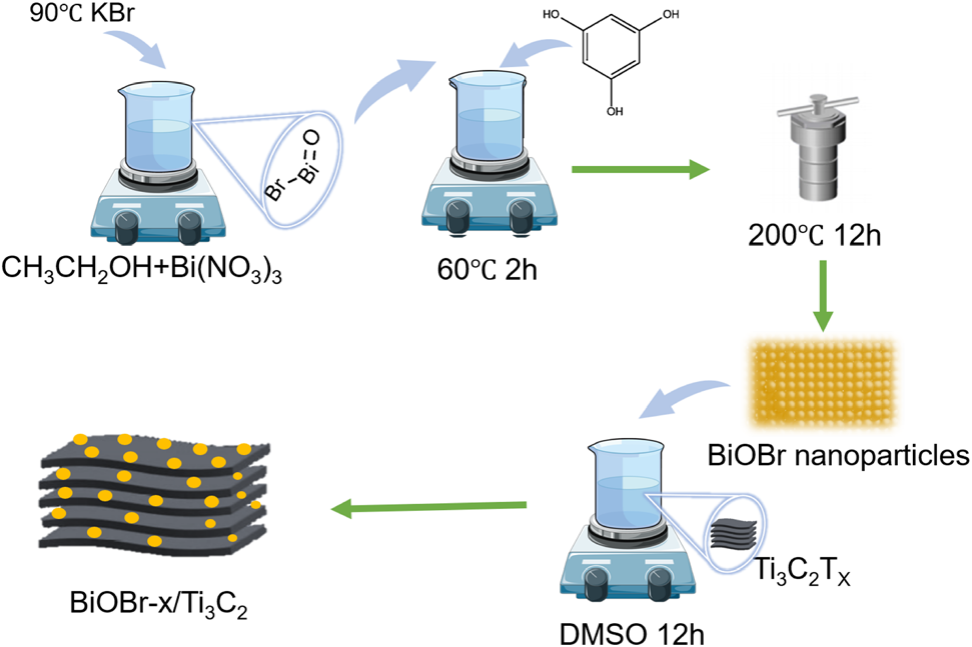
Schematic of the preparation of BiOBr/Ti_3_C_2_.

**Fig. 1:**
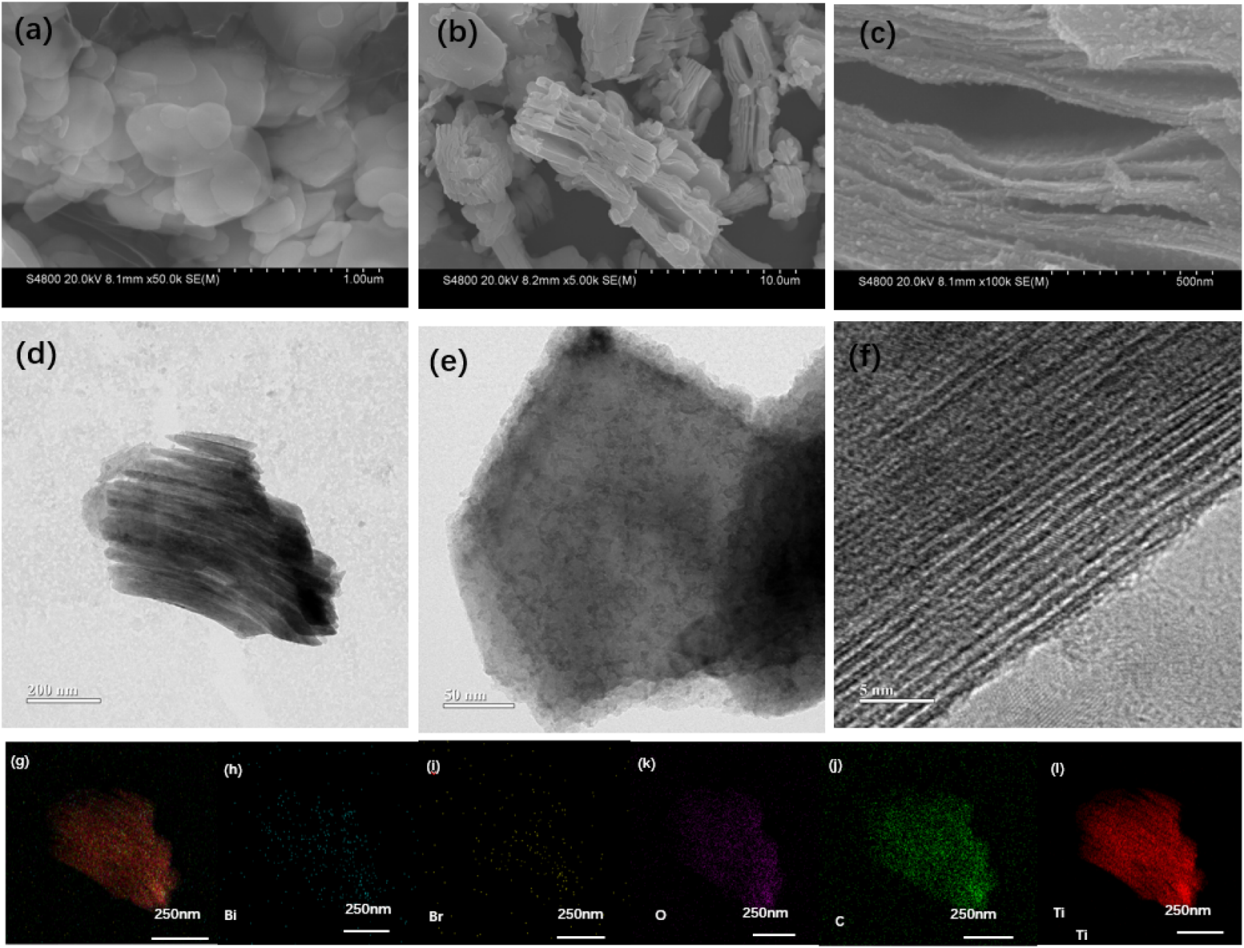
SEM images of (a) BiOBr, (b) Ti_3_C_2_T_*x*_ and (c) BiOBr-10/Ti_3_C_2_; (d) TEM and magnified TEM (e) images of BiOBr-10/Ti_3_C_2_; (f) HRTEM image of BiOBr-10/Ti_3_C_2_; elemental mapping images of (g) overall distribution, (h) Bi, (i) Br, (j) C, (k) O and (l) Ti in BiOBr-10/Ti_3_C_2_.

Scheme 1 illustrates the synthetic strategy of BiOBr/Ti_3_C_2_. The morphological changes and elemental distribution of BiOBr nanoparticles, Ti_3_C_2_T_*x*_, and BiOBr-*x*/Ti_3_C_2_ were observed and compared by SEM and TEM images, as shown in Fig. 1a–l. The preparation of BiOBr/Ti_3_C_2_ was divided into two steps. After heat treatment with distilled water and phloroglucinol,^57^ BiOBr became nanoparticles, which favored adhering to the surface of Ti_3_C_2_T_*x*_. Subsequently, the BiOBr nanoparticles and Ti_3_C_2_T_*x*_ matrix were put into DMSO to make the MXene interlayer open and disperse more evenly. Finally, BiOBr nanoparticles were more easily loaded within the interlayers, and they further formed heterojunctions with MXene Ti_3_C_2_T_*x*_. In the SEM images, pristine BiOBr agglomerates (Fig. 1a) and pristine MXene Ti_3_C_2_T_*x*_ do not show obvious delamination (Fig. 1b). For BiOBr-10/Ti_3_C_2_, DMSO showed a polar effect and increased the distance of the MXene Ti_3_C_2_T_*x*_ layers, so that BiOBr nanoparticles could be loaded and highly dispersed in Ti_3_C_2_T_*x*_ interlayers (Fig. 1c). BiOBr-10/Ti_3_C_2_ retained the accordion shape and exhibited a rough surface (Fig. 1c), indicating the strong combination of BiOBr nanoparticles with Ti_3_C_2_T_*x*_ and the formation of a heterojunction. The TEM images (Fig. 1d and e) show that Ti_3_C_2_T_*x*_ is well-laminated and loaded with ultrafine and uniform BiOBr nanoparticles. In the HRTEM image (Fig. 1f), BiOBr nanoparticles were embedded between the Ti_3_C_2_T_*x*_ lattices, indicating the existence of strong interactions and interfaces. The concave and convex surface of Ti_3_C_2_T_*x*_ provides a good platform for the formation of BiOBr/Ti_3_C_2_ heterojunctions. The elemental mapping images further confirmed the successful binding of BiOBr nanoparticles with Ti_3_C_2_T_*x*_ (Fig. 1g–i). Ti and C are widely distributed throughout the entire region of the composite as the main elements of the matrix, whereas Bi, Br, and O are evenly distributed and have relatively low density. The co-existence and overlapping distribution of the elements indicate a great opportunity to form a heterojunction on the surface of Ti_3_C_2_T_*x*_.

**Fig. 2:**
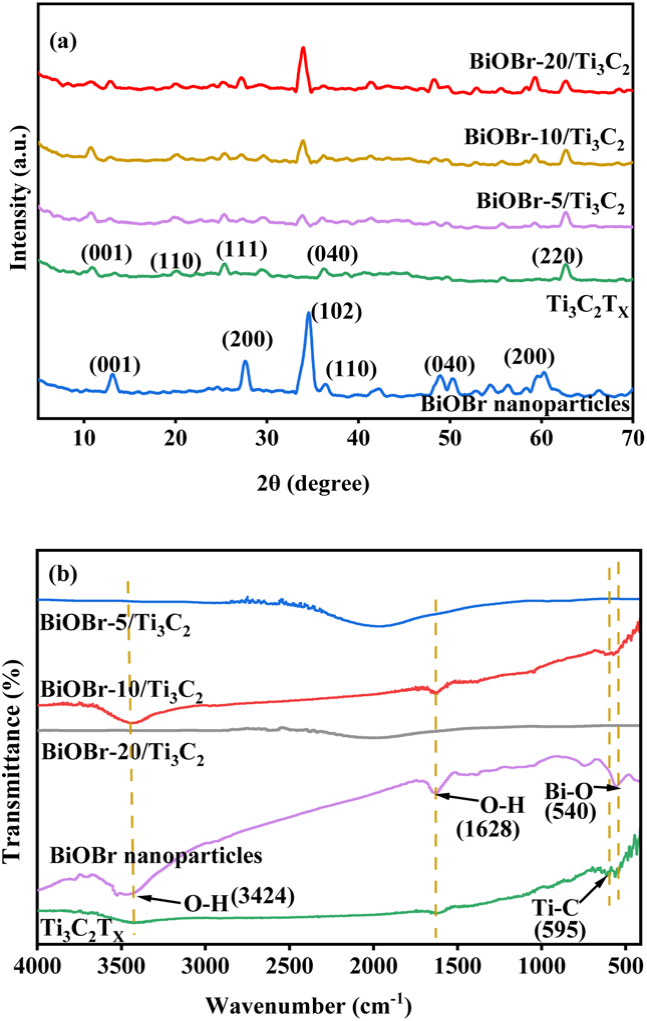
(a) XRD patterns and (b) FT-IR spectra of BiOBr, Ti_3_C_2_T_*x*_ and BiOBr-*x*/Ti_3_C_2_.

XRD and FT-IR spectra were used to investigate the structure of the BiOBr-*x*/Ti_3_C_2_ composites with different BiOBr loads, as shown in Fig. 2a and b, respectively. In Fig. 2a, pure BiOBr shows five peaks at 2 theta of 13.24°, 27.76°, 34.72°, 50.12°, and 60.08°, corresponding to the five crystal planes of (001), (200), (040), (040), and (200), respectively.^52^ Pure Ti_3_C_2_T_*x*_ shows four peaks at 2 theta of 10.06°, 19.72°, 25.2°, and 62.2°, corresponding to the four crystal planes of (001), (110), (111), and (220), respectively.^58^ For the BiOBr-*x*/Ti_3_C_2_ composites, the crystal planes of (001), (200), (040), (040) and (200) become more obvious with the increase of BiOBr loading. The results indicate that BiOBr nanoparticles were successfully loaded onto Ti_3_C_2_T_*x*_, but their size was larger and uneven at higher loading. Ti_3_C_2_T_*x*_ was treated with DMSO to make its layers more dispersed and uniform and to increase the layer spacing. Therefore, the peaks intensity of the Ti_3_C_2_T_*x*_ phase peaks increased with BiOBr loading in the composite, indicating that the Ti_3_C_2_T_*x*_ matrix maintained the original accordion-like layer structure and provided larger layer spacing for BiOBr loading. However, the peak intensity of Ti_3_C_2_T_*x*_ for BiOBr-20/Ti_3_C_2_ did not increase because the Ti_3_C_2_T_*x*_ layers were partially filled by overloaded BiOBr. The obvious delamination of Ti_3_C_2_T_*x*_ and the increase in the contact area with BiOBr nanoparticles increase the tendency to form heterojunctions, which is conducive to the transport of photogenerated charges.

FT-IR spectroscopy was used to compare the variation of the main groups in the BiOBr/Ti_3_C_2_ composites, as shown in Fig. 2b. The FT-IR spectrum of pure Ti_3_C_2_T_*x*_ has a Ti–C corresponding vibration band at 587 cm^−1^.^59^ The wide peaks at 3450 cm^−1^ belong to the O–H tensile vibrations, which may be caused by surface water molecules or terminal hydroxyl groups.^59^ The FT-IR spectrum of pure BiOBr shows a peak at 517 cm^−1^, which is attributed to the symmetric stretching vibration of the Bi–O bond.^60^ In addition, the peaks observed at 1630 and 3430 cm^−1^ in the BiOBr-*x*/Ti_3_C_2_ composites correspond to O–H vibrations of adsorbed water molecules. The BiOBr-*x*/Ti_3_C_2_ composites also show Bi–O and Ti–C vibration peaks, indicating the simultaneous existence of Ti_3_C_2_T_*x*_ and BiOBr phases.

**Fig. 3:**
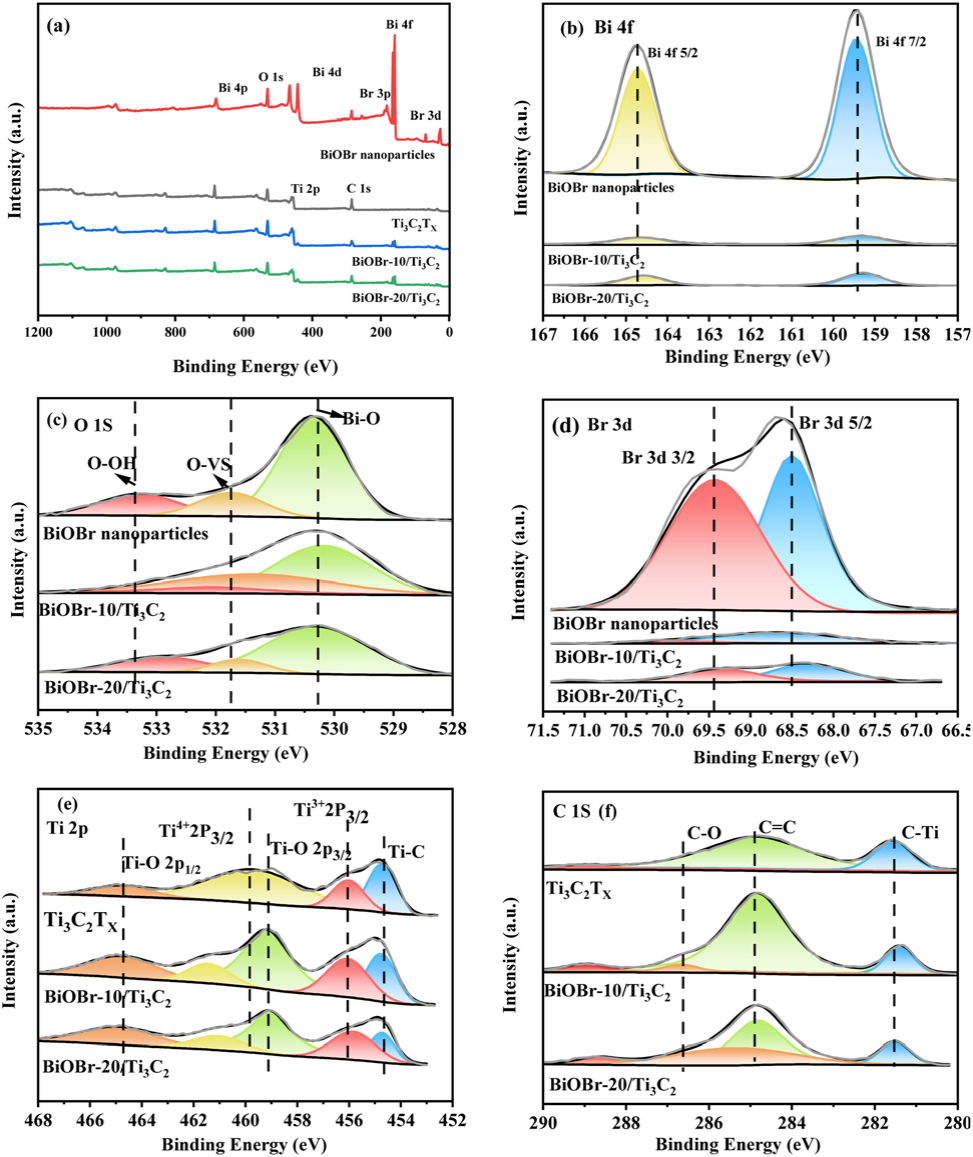
(a) XPS survey spectra; high-resolution XPS spectra of (b) Bi 4f, (c) O 1s, (d) Br 3d, (e) Ti 2p and (f) C 1s for BiOBr, Ti_3_C_2_T_*x*_ and BiOBr-*x*/Ti_3_C_2_.

XPS was employed to investigate the surface elemental states of the photocatalysts, which provided evidence for the successful combination of BiOBr and Ti_3_C_2_T_*x*_. XPS survey spectra and high-resolution XPS spectra of BiOBr, Ti_3_C_2_T_*x*_ and BiOBr-*x*/Ti_3_C_2_ samples are presented in Fig. 3a–f. In Fig. 3a, Ti, C, Bi, O, and Br are detected in the XPS spectra of BiOBr-*x*/Ti_3_C_2_ composites supporting 10 wt% and 20 wt% of BiOBr. In the Bi 4f spectrum shown in Fig. 3b, two distinct peaks with binding energies of 159.5 and 164.7 eV are assigned to Bi 4f_5/2_ and Bi 4f_7/2_, respectively.^61^

In Fig. 3c, each O 1s spectrum can be deconvoluted into three peaks. The peak at 530.4 eV corresponds to lattice oxygen, while the peaks at 531.8 eV and 533.3 eV are attributed to oxygen vacancies (OVs) and chemisorbed oxygen (hydroxyl groups), respectively.^62^ In Fig. 3d, the peaks at 68.5 and 69.4 eV are associated with Br 3d_5/2_ and Br 3d_3/2_, respectively.^61^ In Fig. 3e, the Ti 2p spectrum of each BiOBr-*x*/Ti_3_C_2_ sample can be deconvoluted into five peaks located at 454.7, 456.1, 459.2, 459.8 and 464.8 eV, corresponding to Ti–C, Ti^3+^ (Ti 2p_3/2_), Ti–O (2p_3/2_), Ti^4+^ (Ti 2p_3/2_) and Ti–O (2p_1/2_), respectively.^58,59^ In contrast, the Ti 2p spectrum of pure Ti_3_C_2_T_*x*_ exhibits only four peaks. This difference is primarily attributed to the peak at 459.2 eV, which indicates the formation of Ti–O bonds between the O in BiOBr and the Ti in MXene Ti_3_C_2_T_*x*_. In Fig. 3f, the C 1s spectrum is deconvoluted into four peaks at 281.6, 284.9, and 286.7 eV, corresponding to C–Ti bonds, sp^2^ carbon C=C, and C–O bonds, respectively.^58^ Compared to pure BiOBr, the Bi 4f, O 1s, and Br 3d peaks of the BiOBr-*x*/Ti_3_C_2_ samples exhibited a significant shift toward lower binding energies (Fig. 3b–d), while the Ti 2p peaks shifted toward higher binding energies (Fig. 3e). The observations indicate that electrons are gained during the oxidation process of BiOBr, leading to observed shifts in the binding energies of Bi, O, and Br to lower fields. Simultaneously, Ti and C in the composite material lose electrons, resulting in a shift to higher fields.

### Photoelectric properties of materials

**Fig. 4:**
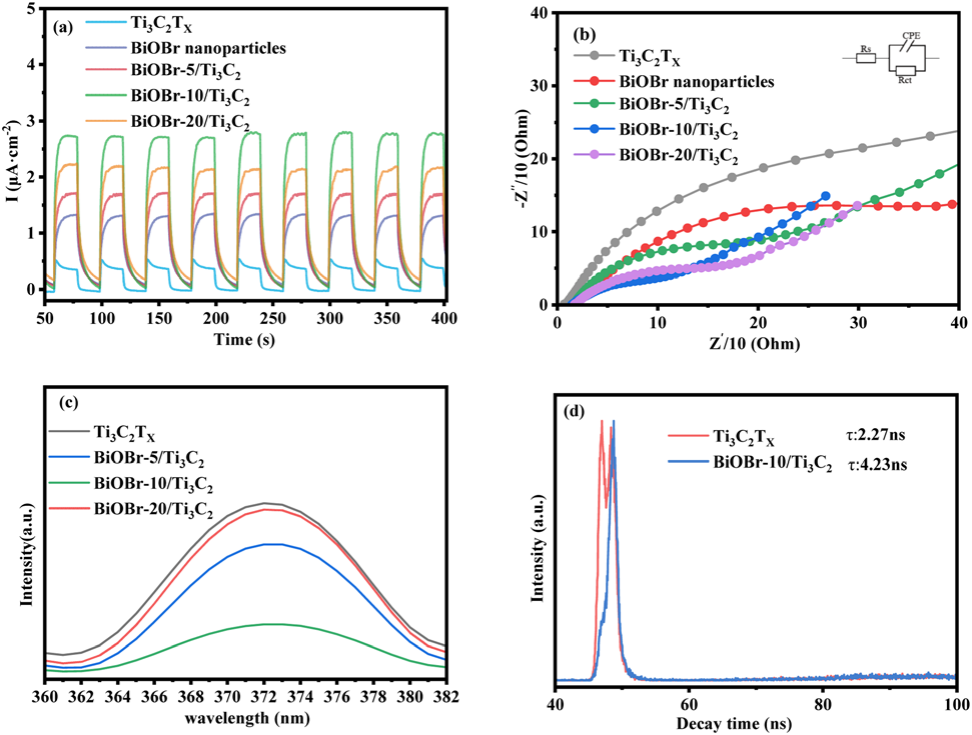
(a) Transient photocurrent density, (b) Nyquist plots, (c) PL spectra and (d) TRPL spectra of Ti_3_C_2_T_*x*_, BiOBr and BiOBr-*x*/Ti_3_C_2_.

The photocurrent and EIS reflect the ability of photo-excited carriers to separate and transport under illumination. Fig. 4a shows a comparison of the photocurrent response of the BiOBr-*x*/Ti_3_C_2_ composites with different BiOBr loadings. Compared with the original Ti_3_C_2_T_*x*_, the transient photocurrent response of a BiOBr/Ti_3_C_2_ sample was significantly increased, indicating that the interfacial charge transfer ability of Ti_3_C_2_T_*x*_ was promoted due to the formation of heterojunctions between the MXene Ti_3_C_2_T_*x*_ and BiOBr nanoparticles. Among the samples, BiOBr-10/Ti_3_C_2_ exhibited the highest photocurrent density owing to its highly dispersed BiOBr nanoparticles and laminated Ti_3_C_2_T_*x*_ layers (Fig. 1c). As a result, the sample can achieve more efficient photogenerated charge separation and transfer, which helps improve its photocatalytic performance. In addition, the photocurrent of each sample shows stability in intensity with time extension, indicating that the composites can provide a stable number of electrons and holes during the irradiation process and further exhibit stable photocatalytic activity. Fig. 4b shows the comparison of the EIS of BiOBr-*x*/Ti_3_C_2_ composites with different BiOBr loadings. As shown in the figure, the order of the capacitance arc semicircle for the samples is that Ti_3_C_2_T_*x*_ > BiOBr > BiOBr-5/Ti_3_C_2_ > BiOBr-20/Ti_3_C_2_ > BiOBr-10/Ti_3_C_2_. The smaller charge transfer impedance between Ti_3_C_2_T_*x*_ layers and the BiOBr nanoparticles is conducive to the separation of e^+^–h^+^ pairs in the photocatalytic process. The capacitance arc semicircle of BiOBr-10/Ti_3_C_2_ is the smallest, indicating that a more efficient interface between BiOBr nanoparticles and Ti_3_C_2_T_*x*_ is constructed, which provides more space for the separation and transfer of e^+^–h^+^ pairs and makes it have the best photocatalytic activity.

The state and the lifetime of active electron–hole pairs were further analyzed by PL and TRPL spectra, respectively. Fig. 4c shows the PL spectra of the photocatalyst samples. The pristine Ti_3_C_2_T_*x*_ shows the strongest emission peak at 373 nm. The emission peak intensity of the BiOBr-*x*/Ti_3_C_2_ sample was lower than that of pristine Ti_3_C_2_T_*x*_. In particular, BiOBr-10/Ti_3_C_2_ shows the weakest emission peak, indicating that appropriately loaded BiOBr nanoparticles reduced the speed of e^+^–h^+^ pairs recombination in Ti_3_C_2_T_*x*_. Fig. 4d shows the TRPL spectra of Ti_3_C_2_T_*x*_ and BiOBr-10/Ti_3_C_2_. The average fluorescence lifetimes of Ti_3_C_2_T_*x*_ and BiOBr-10/Ti_3_C_2_ were 2.27 and 4.23 ns, respectively, which were calculated by eqn (3). The longer the fluorescence lifetime, the more conducive it is to carrier separation, which is conducive to photogenerated carriers participating in the photocatalytic reaction. Based on the photocurrent, EIS, PL and TRPL spectra, it can be concluded that the interfacial electron transfer was improved after the formation of the BiOBr/Ti_3_C_2_ heterojunction.

3 *τ*_a_ = (*A*_1_*τ*_1_^2^ + *A*_2_*τ*_2_^2^)/(*A*_1_*τ*_1_ + *A*_2_*τ*_2_)

where *τ* and *A* are the emission lifetimes of each component and amplitude, respectively.

### Photocatalytic performance of roxarsone degradation

**Fig. 5:**
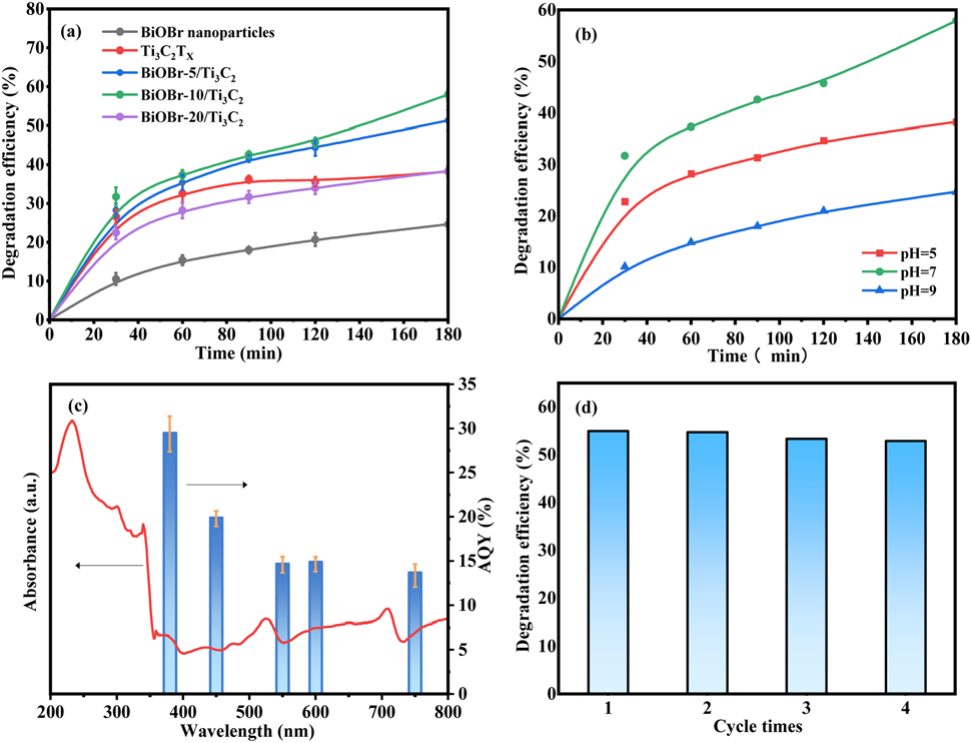
(a) Photocatalytic degradation of roxarsone over different photocatalysts (conditions: 300 W Xe lamp, 0.05 g catalyst, 50 mL 2 mg L^−1^ of roxarsone solution, pH = 7.0); (b) effect of the medium's pH on photocatalytic degradation of roxarsone over BiOBr-10/Ti_3_C_2_ (conditions: 300 W Xe lamp, 0.05 g catalyst, 50 mL 2 mg L^−1^ of roxarsone solution); (c) UV-vis absorption spectrum of BiOBr-10/Ti_3_C_2_ (left axis) and wavelength dependent AQY (%) of photocatalytic degradation of roxarsone (right axis); and (d) recycling ability of BiOBr-10/Ti_3_C_2_ (conditions: 300 W Xe lamp, 0.05 g catalyst, 50 mL 2 mg L^−1^ of roxarsone solution, pH = 7.0, 3 h).

The photocatalytic activity of the samples was evaluated by the photocatalytic degradation of roxarsone under visible light. Fig. 5a shows a comparison of the photocatalytic degradation of roxarsone by different photocatalysts. Pure Ti_3_C_2_T_*x*_ exhibits basal photocatalytic activity for roxarsone degradation, achieving 38.21% removal for 3 h, and 1.06 mg g_cat._^−1^ h^−1^ of degradation efficiency at 0.5 h, which is almost twice as high as that of pure BiOBr. Compared with pure Ti_3_C_2_T_*x*_, the BiOBr-*x*/Ti_3_C_2_ sample exhibits a significant enhancement in the photocatalytic activity for roxarsone degradation. The activity order of the BiOBr-*x*/Ti_3_C_2_ samples was BiOBr-10/Ti_3_C_2_ > BiOBr-5/Ti_3_C_2_ > BiOBr-20/Ti_3_C_2_. BiOBr-10/Ti_3_C_2_ as the optimal sample achieved 57.92% removal for 3 h, and 1.27 mg g_cat._^−1^ h^−1^ of degradation efficiency at 0.5 h. The results indicate that modification of an appropriate amount (10 wt%) of BiOBr for Ti_3_C_2_T_*x*_ can promote the separation of photogenerated carriers and the transfer of electrons to effectively participate in the photocatalytic reaction. However, BiOBr-20/Ti_3_C_2_ exhibited lower photocatalytic degradation ability for roxarsone than pure Ti_3_C_2_T_*x*_. As discussed above, excess BiOBr in the composite results in the coverage of the active sites within the Ti_3_C_2_T_*x*_ layers, which lowers the photogenerated carrier density and restrains the photoelectron transfer and the separation of e^+^–h^+^ pairs. Fig. 5b shows the effect of medium pH on the photocatalytic degradation of roxarsone. The most efficient degradation of roxarsone can be achieved in a neutral environment. Acidic condition inhibits the immobilization of As^5+^ products, both reducing the degradation efficiency and increasing the risk of secondary pollution. Under alkaline conditions, As^5+^ tends to form soluble arsenate with OH^−^, and the active sites of photocatalysts are poisoned, enhancing arsenic migration. Fig. 5c shows the AQY values for the photocatalytic degradation of roxarsone by BiOBr-10/Ti_3_C_2_ at different wavelengths. The AQY values at 380, 450, 550, 600, and 750 nm were calculated to be 29.5%, 19.9%, 14.7%, 14.9%, and 13.7%, respectively. The trend of the AQY value agreed well with the diffuse reflectance spectrum. Fig. 5d shows the recyclability of BiOBr-10/Ti_3_C_2_ for the photocatalytic degradation of roxarsone. The spent photocatalyst in each cycle was sufficiently washed with water and ethanol. After treatment, the photocatalyst exhibited good recyclability and could be reused for 4 cycles.

### Photocatalytic mechanism

**Fig. 6:**
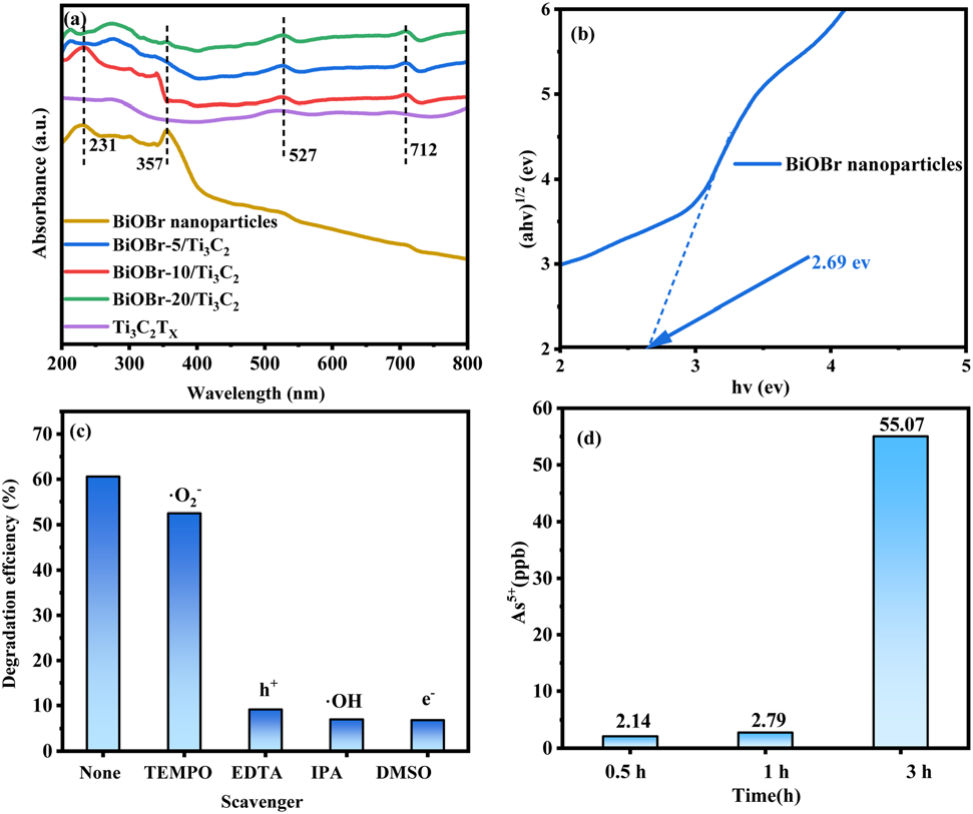
(a) UV-vis absorption spectra of BiOBr, Ti_3_C_2_T_*x*_ and BiOBr-*x*/Ti_3_C_2_; (b) Kubelka–Munk plot of BiOBr; (c) photocatalytic degradation of roxarsone over BiOBr-10/Ti_3_C_2_ (conditions: 300 W Xe lamp, 0.05 g catalyst, 50 mL 2 mg L^−1^ of roxarsone solution, pH = 7, 1 h); (d) final product detection for roxarsone degradation at different times (conditions: 300 W Xe lamp, 0.05 g catalyst, 50 mL 2 mg L^−1^ of roxarsone solution, pH = 7.0).

UV-vis diffuse reflectance spectra were used to investigate the band structure of the BiOBr/Ti_3_C_2_ heterojunction, as shown in Fig. 6a. Pure BiOBr shows absorption bands at 231 and 358 nm, and the absorption range does not exceed 450 nm, which limits the use of the visible part of solar energy. Pure Ti_3_C_2_T_*x*_ exhibits stronger absorption in the ultraviolet region (200–400 nm) and reduced absorption in the visible region (500–800 nm). With the increase of BiOBr content, the absorption strength of the BiOBr-*x*/Ti_3_C_2_ composite is increased in the visible region, indicating that BiOBr combined with Ti_3_C_2_T_*x*_ effectively improves the light absorption range and ability of MXene. Based on Fig. 6a, the bandgaps (*E*_g_) of the samples were determined by the transformation of the Kubelka–Munk function to the UV-vis diffuse reflectance.^58,63^Fig. 6b shows the (*ahv*)^1/2^*vs.* (*hv*) plot for BiOBr nanoparticles. The bandgap of BiOBr was estimated to be 2.69 eV. The values of *E*(H_2_O/˙OH) and *E*(O_2_/˙O^2−^) were 2.8 and −0.33 eV,^64^ respectively. In addition, the *E*_VB_ position of BiOBr was approximately 3.02 eV, whereas the *E*_CB_ position was not more negative than the *E*(O_2_/˙O^2−^) value.^65^ Therefore, ˙OH was significantly generated and contributed to the redox reaction instead of ˙O^2−^.

**Scheme 2:**
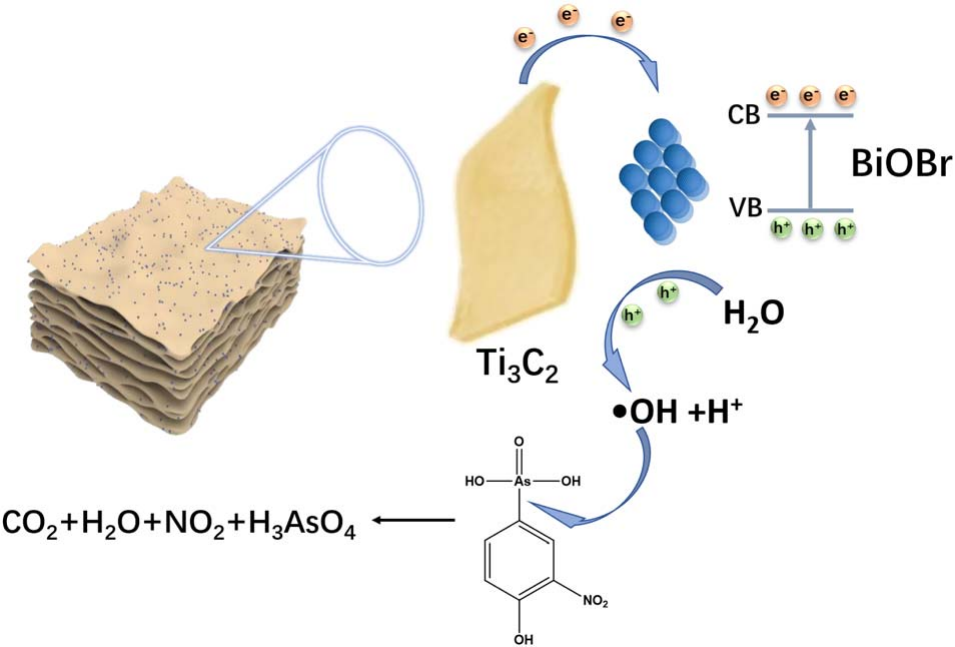
Proposed mechanism for the photocatalytic degradation of roxarsone over BiOBr/Ti_3_C_2_.

To elucidate the mechanism of the photocatalytic degradation of roxarsone, active species capture experiments were performed with EDTA, DMSO, IPA and TEMPOL as scavengers of h^+^, e^−^, ˙OH and ˙O_2_^−^, respectively, as shown in Fig. 6c. The ˙OH and h^+^ scavengers (DMSO, EDTA) had obvious inhibitory effects on the degradation of roxarsone, indicating that ˙OH is an active substance essential for the degradation of roxarsone, while h^+^ plays an important role in ˙OH generation. The inhibition by ˙O_2_^−^ scavenger (TEMPOL) was very weak, indicating that ˙O_2_^−^ is not the main active substance for the photocatalytic degradation of roxarsone. Although the scavenger (DMSO) has the most remarkable inhibitory effect, the electrons only contribute to the separation of e^−^–h^+^ pairs to produce more holes for roxarsone degradation, instead of direct participation in the reaction. The residual inorganic arsenic forms in the liquid phase were confirmed by LC-ICP-MS to analyze the reaction details and assess the safety after water treatment. As shown in Fig. 6d, As^5+^, as the final product of roxarsone, can be detected after each reaction period, and its concentration increases with the reaction time. From the residual roxarsone concentration (2–50 ppb, Fig. 6d), it can be assessed that the treated water by the present system was at a safe level, according to the specified level (0.01 mg L^−1^) by the World Health Organization (WHO). Based on these findings, we propose the mechanism for the photocatalytic degradation of roxarsone over BiOBr/Ti_3_C_2_, as illustrated by eqn (4)–(6) and Scheme 2. Upon absorption of light energy by BiOBr/Ti_3_C_2_, electrons are excited from the VB to the CB, which generates abundant holes in the VB of the photocatalyst (eqn (4)). The holes oxidize water molecules to generate abundant hydroxyl radicals (˙OH) (eqn (5)).^66^ Finally, ˙OH participates in the mineralization and oxidation of roxarsone to form inorganic products (eqn (6)).

4 BiOBr/Ti_3_C_2_ + *hν* → h^+^ + e^−^

5 h^+^ + H_2_O → ˙OH + H^+^

6 C_6_H_6_AsNO_6_ + ˙OH → CO_2_ + H_2_O + NO_2_ + H_3_AsO_4_

## Conclusions

In summary, BiOBr nanoparticles were successfully loaded onto MXene Ti_3_C_2_T_*x*_ to form a heterostructure for the photocatalytic degradation of roxarsone. The proposed construction strategy effectively addressed the issue of low photocatalytic efficiency of Ti_3_C_2_T_*x*_ in environmental protection areas. The uniform dispersion of BiOBr nanoparticles on the surface of Ti_3_C_2_T_*x*_ layers promoted the formation of an interface between the two phases, significantly enhancing the electron transport capability, electron donor density, and the separation efficiency of electron–hole pairs. The optimized BiOBr/Ti_3_C_2_ material exhibited excellent photocatalytic performance for roxarsone degradation, along with an AQY of 29.5% and good reusability for at least 4 cycles. A series of in-depth mechanism studies proposed that h^+^ and ˙OH in the VB of the BiOBr/Ti_3_C_2_ system are involved in the main pathway of the oxidative mineralization of roxarsone. This work is expected to elucidate more feasible and scalable photocatalytic systems for organoarsenic-contaminated wastewater treatment with high efficiency and sustainability.

## Author contributions

Yaxin Guo: methodology, data curation, software, and writing – original draft. Ya-Nan Wang: formal analysis and methodology. Jinsong Peng: project administration. Haiyan Song: project administration, methodology, data curation, writing – original draft, and writing – review & editing. Chunxia Chen: project administration and methodology.

## Conflicts of interest

There are no conflicts to declare.

## Data Availability

Data will be made available upon request.
